# Correlations among PPAR**γ**, DNMT1, and DNMT3B Expression Levels and Pancreatic Cancer

**DOI:** 10.1155/2012/461784

**Published:** 2012-08-08

**Authors:** Valerio Pazienza, Francesca Tavano, Giorgia Benegiamo, Manlio Vinciguerra, Francesca Paola Burbaci, Massimiliano Copetti, Fabio Francesco di Mola, Angelo Andriulli, Pierluigi di Sebastiano

**Affiliations:** ^1^Division and Laboratory of Gastroenterology Unit, “Casa Sollievo della Sofferenza” IRCCS Hospital, Viale dei Cappuccini n.1, 71013 San Giovanni Rotondo, Italy; ^2^Division of Abdominal Surgery, “Casa Sollievo della Sofferenza” IRCCS Hospital, 71013 San Giovanni Rotondo, Italy; ^3^The Foundation for Liver Research, c/o The Institute of Hepatology, 69-75 Chenies Mews, London WC1E 6HX, UK; ^4^Biostatistic Unit, “Casa Sollievo della Sofferenza” IRCCS Hospital, 71013 San Giovanni Rotondo, Italy

## Abstract

Emerging evidence indicates that peroxisome proliferator-activated receptor **γ** (PPAR**γ**) and DNA methyltransferases (DNMTs) play a role in carcinogenesis. In this study we aimed to evaluate the expression of PPAR**γ**, DNMT1, and DNMT3B and their correlation with clinical-pathological features in patients with pancreatic cancer (PC), and to define the effect of PPAR**γ** activation on DNMTs expression in PC cell lines. qRT-PCR analysis showed that DNMT3B expression was downregulated in tumors compared to normal tissues (*P* = 0.03), whereas PPAR**γ** and DNMT1 levels did not show significant alterations in PC patients. Expression levels between PPAR**γ** and DNMT1 and between DNMT1 and DNMT3B were highly correlated (*P* = 0.008 and *P* = 0.05 resp.). DNMT3B overexpression in tumor tissue was positively correlated with both lymph nodes spreading (*P* = 0.046) and resection margin status (*P* = 0.04), and a borderline association with perineural invasion (*P* = 0.06) was found. Furthermore, high levels of DNMT3B expression were significantly associated with a lower mortality in the whole population (HR = 0.485; 95%CI = 0.262–0.895, *P* = 0.02) and in the subgroup of patients without perineural invasion (HR = 0.314; 95%CI = 0.130–0.758; *P* = 0.01), while such association was not observed in patients with tumor invasion into perineural structures (*P* = 0.70). In conclusion, *in vitro* and *in vivo* PPAR**γ** and DNMTs appear interrelated in PC, and this interaction might influence cell phenotype and disease behavior.

## 1. Introduction

Pancreatic cancer (PC) is ranked as the fourth leading cause of cancer-related deaths worldwide [1, 2]. It is highly aggressive and resistant to chemotherapy, and our inability to detect it at an early stage and the lack of effective systemic therapies are responsible for nearly identical incidence and mortality rates [3, 4]. More effective treatments and/or development of novel strategies are needed to improve the prognosis for patients with PC.

The peroxisome proliferator-activated receptors (PPARs) belong to the nuclear receptor superfamily and are considered master regulators of lipid and glucose metabolism by transducing metabolic and nutritional signals into transcriptional responses [5, 6]. Three subtypes of PPARs are known: PPAR*α*, PPAR*δ*, and PPAR*γ* [7]. The latter has been implicated in the pathology of numerous diseases including obesity, diabetes, atherosclerosis, and cancer. PPAR*γ* ligands induce differentiation of liposarcoma cells and have a variety of antitumor effects also in pancreatic cancer cells [8]. The availability of such high-affinity ligands has facilitated the study of the signalling pathways through which PPAR*γ* regulates metabolic processes, which are regulated also by epigenetic events. The mechanisms underlying epigenetic modulation mediated by PPARs remain to be fully explored. DNA methyltransferases (DNMTs) are critical in epigenetic events through the addition of methyl groups to DNA [9, 10]. Maintenance of methylation pattern is achieved by DNMT1 function [11] during DNA replication while new or *de novo* methylation is primarily catalyzed by DNMT3a and DNMT3b [12]. Whether and how PPARs modulate epigenetic events remain to be fully explored. In this paper, we sought to examine mRNA levels of PPAR*γ* and DNMT1 and 3B in a cohort of PC patients and to correlate the findings with clinical-pathologic features, including patient survival, and to evaluate whether pharmacological modulation of PPAR*γ* could influence the expression of DNMTs in PC cell lines.

## 2. Material and Methods

### 2.1. Patients and Tissues Samples Preparation

A cohort of 30 matched pairs of tumour and adjacent normal tissue samples were collected from patients undergoing pancreatic resection at the Department of Surgery, “Casa Sollievo della Sofferenza” Hospital, IRCCS, San Giovanni Rotondo, Italy between October 2007 and June 2011. Written informed consent was obtained before collection of tissues from patients. The final diagnosis of pancreatic ductal adenocarcinoma was ascertained in all patients by histological examinations. At the last followup, 18 (60%) patients were still alive and 12 (40%) patients had died. Demographics and clinical characteristics of patients are shown in Table 1.

Tissue specimens were immediately frozen in liquid nitrogen, and stored at −80°C until RNA extraction. Cancer cellularity was enriched by cryostat sectioning and dissection of most cellular areas.

### 2.2. Cell Culture and Treatment

BxPC3, CF-PAC, MiaPaca, and Panc1 cells were cultured at 37°C in 5% CO_2_ atmosphere in DMEM medium supplemented with 10% fetal bovine serum (FBS), 100 U/mL penicillin and 100 ng/mL streptomycin (Invitrogen Life Technologies, Milan, Italy) while CFPAC and MiaPaca were maintained in RPMI medium (Invitrogen Life Technologies, Milan, Italy). Treatment with rosiglitazone (purchased from Cayman Chemicals) was performed at different time points (24 hours and 48 hours) and at different concentration (5 *μ*M and 15 *μ*M).

### 2.3. Quantitative Real-Time PCR (qRT-PCR)

Total RNA was extracted from 30 PC fresh frozen specimens and from different pancreatic cancer cells (BxPC3, CFPAC, MiaPaca, and Panc1) using the RNeasy Mini Kit (Qiagen S.P.A. Milano Italy) and subsequently digested by DNase I. cDNA was synthesized from 50 ng total RNA and quantitative real-time PCR was performed using QuantiFast Sybr Green PCR kit following the one-step protocol. For real-time RT-PCR, we used the following SYBR Green QuantiTect Primer purchased from Qiagen: human PPAR*γ* (QT00029841) DNMT1 (QT00034335) and DNMT3B (QT00032067). Reactions were set up in 96-well plates using a 7700 real-time PCR System (Applied Biosystems, Foster City, CA) and all samples were assayed in triplicate. Optical data obtained were analyzed using the default and variable parameters available in the SDS software package (version 1.9.1; Applied Biosystems, Foster City, CA). Expression levels of target gene were normalized using the housekeeping control genes: TATA binding protein (TBP, QT00000721) as previously performed [14].

### 2.4. Statistics

Demographic, clinical, and genetic characteristics were reported as median and interquartile range (*Q*1–*Q*3). Gene expression up- or downregulation was tested by using the one-sample Wilcoxon signed-rank test. Group comparisons were performed using the Pearson, chi-square test and the Mann-Whitney *U* test for categorical and continuous variables, respectively. Correlations between continuous variables were assessed using the *r* Spearman coefficient. Time-to-death analyses were performed using Cox proportional hazards regression models and risks were reported as hazards ratios (HR) along with their 95% confidence intervals (95% CI). Overall survival was defined as the time elapsed between surgery and death. For subjects who did not experience the event, time variable was censored at the time of the last available follow-up time. In the time to death analysis, genes' expressions were logarithm-transformed to respect Cox model's linearity assumption. Kaplan-Meier curves were also reported for display purposes. All statistical analyses were performed using SAS version 9.1.3 (SAS Institute, Cary, NC, USA). As for cell experiments, results were expressed as means ± SD. For statistical comparison, significance was evaluated using the Student *t* test. Values of *P* < 0.05(*) and *P* < 0.005(**) or *P* < 0.001 (***) were considered statistically significant.

## 3. Results

### 3.1. PPAR*γ*, DNMT1 and 3B Expression in Pancreatic Cancer Biopsies

**Figure 1:**
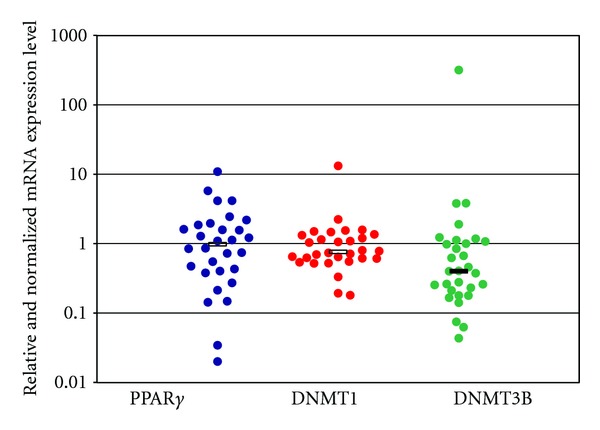
PPAR*γ*, DNMT1, and DNMT3b mRNA expression levels in tissues from 30 patients with pancreatic ductal Adenocarcinoma (PDAC). Each blot indicates the relative expression of genes in tumour compared to normal tissue, after normalization to the endogenous GAPDH. Values greater than 1 indicate gene overexpression in inflamed tissue. For each gene, the median expression levels observed in patients group were indicated by the horizontal black bars. Relative expression values are reported in log scale (*y*-axis).

Relative expression levels of PPAR*γ*, DNMT1, and DNMT3B mRNA in tissue samples from 30 PC patients are presented in Figure 1. Looking at median levels of gene expression in tumor compared to adjacent nontumor tissues, the DNMT3B mRNA expression was downregulated (median = 0.4, *Q*1–*Q*3 = 0.22–1.05, *P* = 0.03), while PPAR*γ* and DNMT1 mRNA levels were not significantly altered (PPAR*γ*: median = 0.98, *Q*1–*Q*3 = 0.41–1.8, *P* = 0.54; DNMT1: median = 0.76; *Q*1–*Q*3 = 0.61–1.29, *P* = 0.4). Analysis of the association among PPAR*γ*, DNMT1, and DNMT3B mRNA levels showed that PPAR*γ* expression levels were positively correlated with DNMT1 expression levels in PC patients (*r* = 0.48, *P* = 0.008), but not with DNMT3B expression levels (*r* = −0.20, *P* = 0.30). A significant correlation between DNMT1 and DNMT3B expression levels in patients with PC was observed, (*r* = 0.36, *P* = 0.053).

### 3.2. Correlation between PPAR*γ*, DNMT1, and DNMT3B mRNA Levels with Clinical and Pathological Features

**Figure 2:**
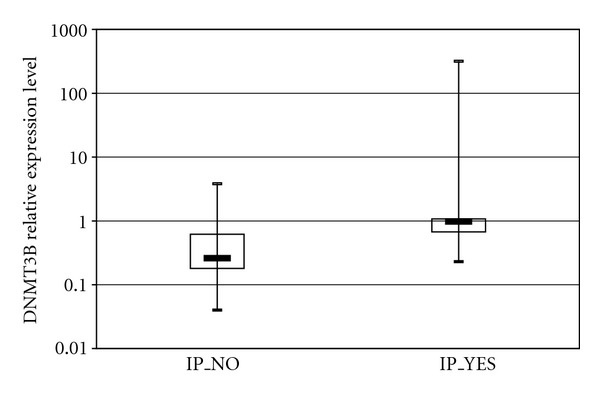
Association of DNMT3b expression levels with perineural invasion (IP) in patients with pancreatic ductal adenocarcinoma (PDAC). Patients were stratified according to IP status (No versus Yes). Each box highlights median, interquartile range (*Q*1–*Q*3) and lower and upper adjacent values (vertical bars) for each subjects group. The upper and lower boundaries of the boxes define the quartiles, 75% and 25% percentiles, respectively, and the black bar represents the median value. Relative expression values are reported in log scale (*y*-axis).

**Figure 3:**
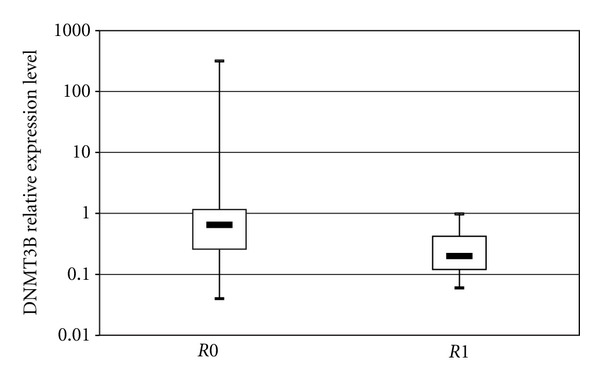
Association of DNMT3b expression levels with resection margins in patients with pancreatic ductal adenocarcinoma (PDAC). Patients were stratified according to resection margin status (*R*0 versus *R*1). Each box highlights median, interquartile range (*Q*1–*Q*3) and lower and upper adjacent values (vertical bars) for each subjects group. The upper and lower boundaries of the boxes define the quartiles, 75% and 25% percentiles, respectively, and the black bar represents the median value. Relative expression values are reported in log scale (*y*-axis).

When PC patients were stratified according to their clinical phenotypes, PPAR*γ* expression levels were unrelated to the considered demographic and clinical features, and DNMT1 showed only a trend of increased expression towards tumors of lower grades of differentiation (*G1*: median = 0.55, *Q*1–*Q*3 = 0.45–0.57; *G2*: median = 0.87, *Q*1–*Q*3 = 0.62–1.47; *G3*: median = 0.94, *Q*1–*Q*3 = 0.75–1.29; *P* = 0.06). Conversely, DNMT3B expression levels were associated with important prognostic variables in PC patients. In details, expression of DNMT3B in tumor tissues was directly related with lymph node ratio, expressed as total involved lymph nodes over total number of resected lymph nodes (*r* = 0.37, *P* = 0.046). Furthermore, DNMT3B expression levels showed a borderline association with perineural invasion in PC patients: DNMT3B levels were higher in patients with evidence of perineural invasion (median = 0.98, *Q*1–*Q*3 = 0.67–1.07) than in those without tumor invasion into neural structures (median = 0.26, *Q*1–*Q*3 = 0.18–0.62), *P* = 0.06 (Figure 2). In addition, DNMT3B expression was higher in patients with resection margins free of tumor cells (*R*0: median = 0.65, *Q*1–*Q*3 = 0.26–1.16), than in those with evidence of tumor infiltration on resected margins (*R*1: median = 0.20, *Q*1–*Q*3 = 0.12–0.42), *P* = 0.04 (Figure 3).

### 3.3. Survival Analysis

**Figure 4:**
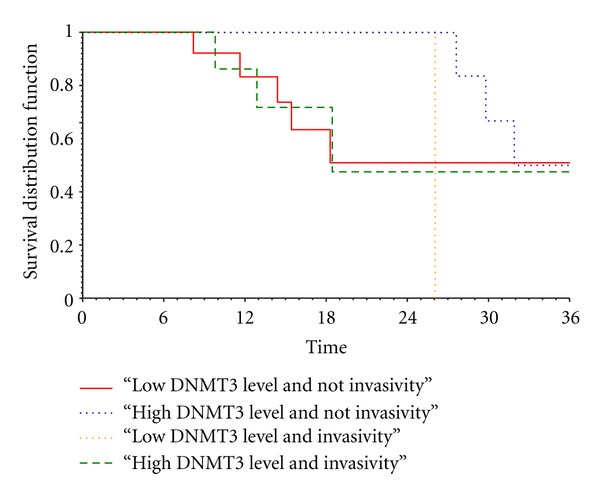


In the time-to-death analysis, DNMT3B expression levels were logarithmic-transformed to accomplish with the linearity assumption of the Cox model. At univariate analysis of the 30 PC patients, DNMT3B high expression levels were associated with lower mortality with an HR= 0.485 (95% CI = 0.262–0.895, *P* = 0.02). In addition, a significant interaction between DNMT3B expression levels and perineural invasion was also observed (*P* = 0.05). In details, in the subgroup of 21 patients without evidence of perineural invasion high DNMT3B expression levels were related with longer survival (HR = 0.314; 95% CI = 0.130–0.758; *P* = 0.01). Conversely, such an effect was not observed in the subgroup of 9 patients with tumour invasion into perineural structures (HR = 0.879; 95%CI = 0.466–1.655; *P* = 0.70) (Figure 4).

### 3.4. PPAR*γ*, DNMT1, and 3b in Pancreatic Cancer Cell Lines

**Figure 5:**
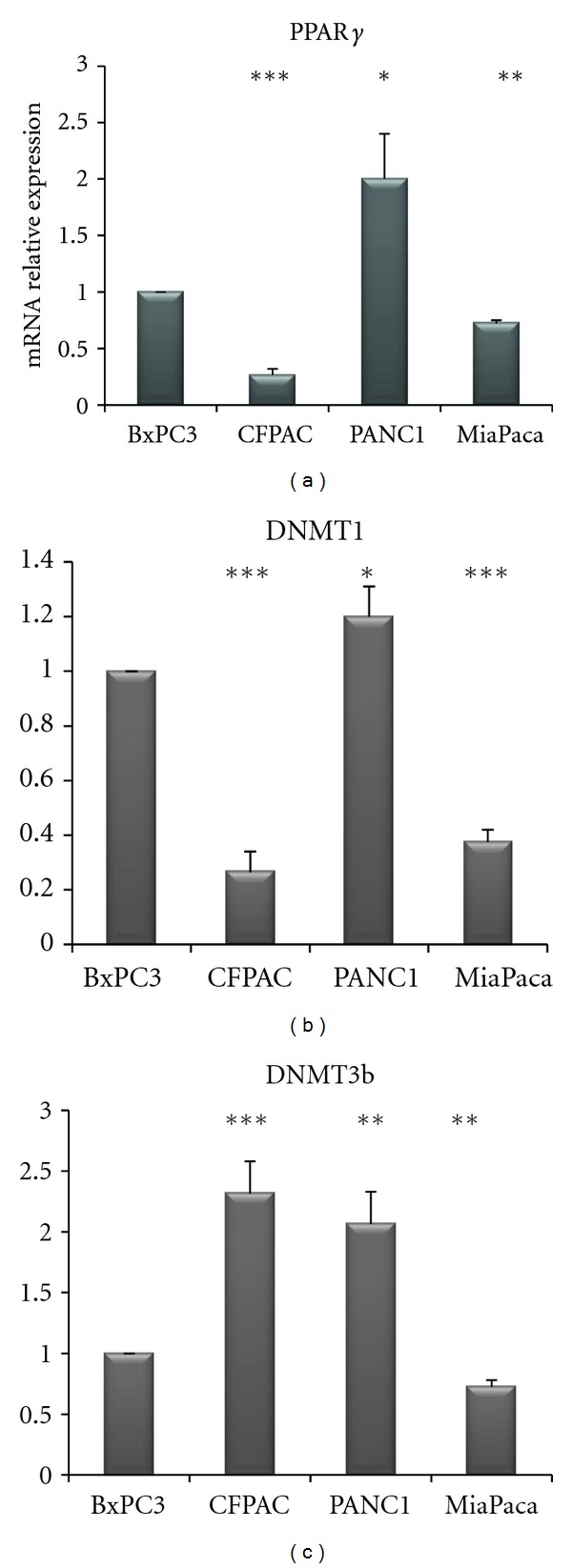
Quantitative real-time PCR. mRNA relative expression levels of PPAR*γ*, DNMT1, and 3B in four different pancreatic cancer cell lines.

In order to corroborate previous findings, we then analyzed PPAR*γ*, DNMT1, and 3B mRNA levels in four different pancreatic cancer cell lines. As shown in Figure 5, PANC-1 cells displayed higher levels of PPAR*γ* as compared to the other cell lines, whilst CFPAC and MiaPaca cells presented lower mRNA levels of PPAR*γ*. A similar pattern was observed for DNMT1 expression. DNMT3B display higher levels in CFPAC and PANC-1cells, while it was downregulated in BxPC3 and MiaPaca cells.

### 3.5. Effect of Rosiglitazone Treatment on DNMTs Expression in Pancreatic Cancer Cell Lines

**Figure 6:**
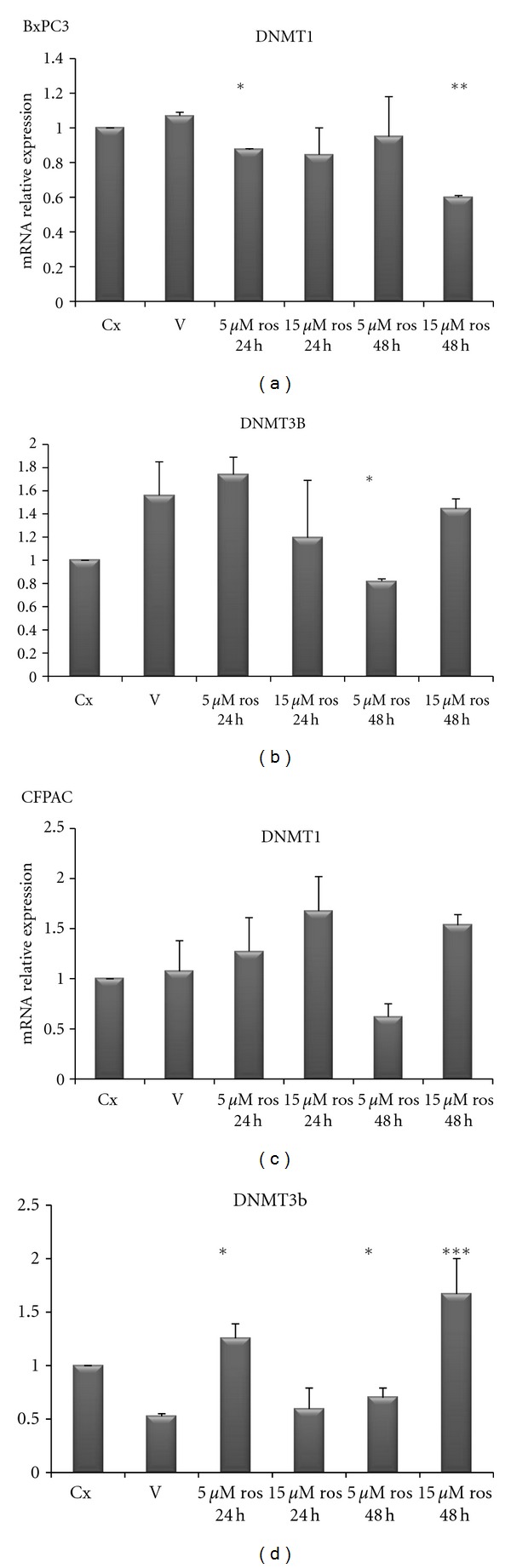
Quantitative real-time PCR. mRNA relative expression levels of DNMT1 and 3B in BxPC3 and CFPAC pancreatic cancer cell lines upon treatment with rosiglitazone at the indicated concentrations and time points.

**Figure 7:**
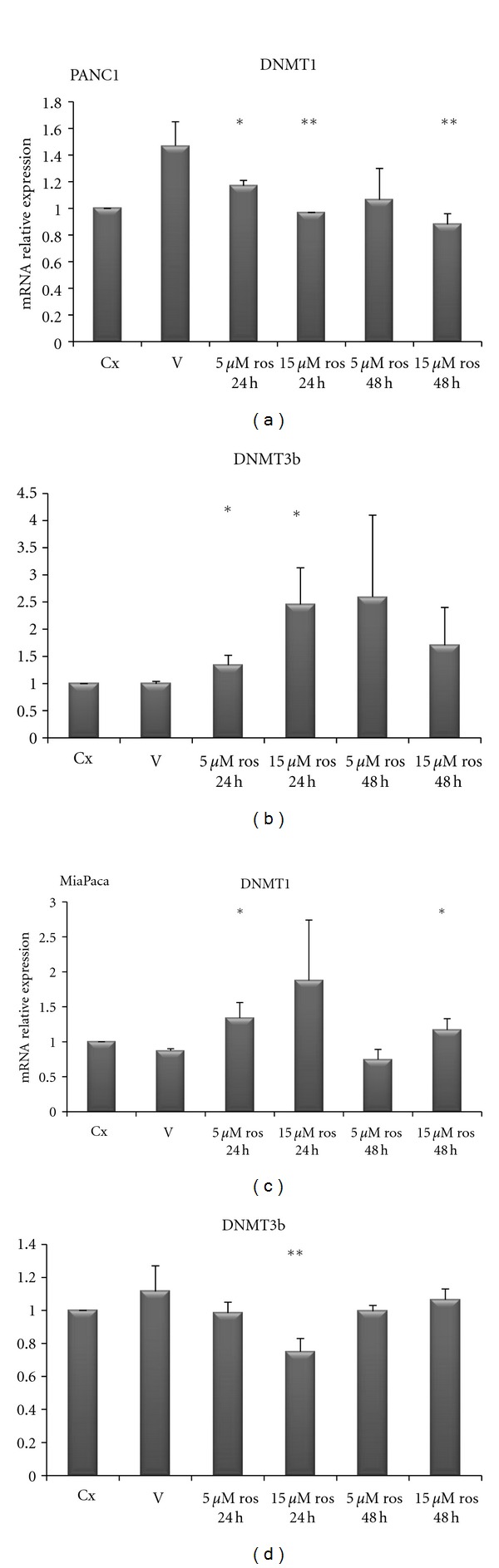
Quantitative real-time PCR. mRNA relative expression levels of DNMT1 and 3B in PANC1 and MiaPaca pancreatic cancer cell lines upon treatment with rosiglitazone at the indicated concentrations and time points.

In order to test whether PPAR*γ* and DNMTs were correlated each other, we used a pharmacological approach by treating PC cells with rosiglitazone (a PPAR*γ* agonist): upon rosiglitazone challenge different expression patterns in different PC cell lines were observed. As shown in Figure 6, in BxPC3 cells rosiglitazone treatment at 15 *μ*M concentration for 48 h decreased DNMT1 expression (*P* < 0.0001) while DNMT3B resulted downregulated after treatment with rosiglitazone at 5 *μ*M for 48 h as compared to vehicle treatment (*P* = 0.003). As for CFPAC cells (Figure 6), rosiglitazone challenge did not influence DNMT1 expression, whereas DNMT3B resulted increased upon treatment with rosiglitazone at 5 *μ*M for 24 h and for 48 h (*P* = 0.0007 and *P* = 0.03, resp.) and after treatment with rosiglitazone at 15 *μ*M for 48 h (*P* = 0.03). In PANC1 cells (Figure 7) DNMT1 was decreased after rosiglitazone treatment at 5 *μ*M for 24 h (*P* = 0.047) and 48 h (*P* = 0.07), and at 15 *μ*M for 24 h (*P* = 0.008) and 48 h (*P* = 0.006). Regarding DNMT3B, rosiglitazone treatment induced a significant upregulation at 5 *μ*M and  15 *μ*M for 24 h (*P* = 0.03 and *P* = 0.02) but not significant at 5 *μ*M and 15*μ*M for 48 h (*P* = 0.139 and *P* = 0.153 resp.).

As for MiaPaca cells (Figure 7), DNMT1 resulted increased after ros treatment at 5 *μ*M and 15 *μ*M for 24 h (*P* = 0.02 and *P* = 0.039 resp.), but this overexpression was not evident after 48 h treatment 5 *μ*M (*P* = 0.21) being still present after 48 h at 15 *μ*M concentration (*P* = 0.003). As for DNMT3B we observed a reduced expression after 48 h treatment with 5 *μ*M of rosiglitazone (*P* = 0.02).

## 4. Discussion

Emerging evidence indicates that PPAR*γ* plays a role in the pathogenesis of several pathological processes such as diabetes, obesity, atherosclerosis, and cancer [15]. Recent scientific reports suggest that the modulation of PPAR*γ* activity may be of therapeutic value in PC [16–19]. PPARs activators might be an ideal combination partner in therapeutic settings where the inhibition of tumour-protecting proteins may be relevant to overcome treatment resistance [20]. Kristiansen et al. [21] demonstrated that PPAR*γ* mRNA and protein expression were upregulated in pancreatic ductal adenocarcinoma and might serve as prognostic marker for this disease. In the present investigation we analyzed mRNA levels of PPAR*γ*, DNMT1, and DNMT3B in PC patients in order to assess correlations among these factors and their associations with some clinical and pathological features of patients. We did not find an altered PPAR*γ* expression in tumor as compared to normal matched tissues, but PPAR*γ* expression showed a positive correlation with DNMT1 but not with DNMT3B expression. In addition, mRNA expression of the latter two genes was highly related. DNMT3B expression was significantly downregulated in tumors as compared to normal matched tissue, and positively associated to lymph node ratio and margin status. Peng et al. [22] suggested that increased DNMT1 protein expression participates in multistage pancreatic carcinogenesis from a precancerous stage to the malignant transition of ductal carcinomas and may be a biological predictor of poor prognosis. In our cohort, we found that DNMT3B expression displayed lower levels in noninvasive compared to invasive tumors. These data were in agreement with the *in vitro* findings showing that DNMT3B levels were higher in PANC1 and CFPAC cells whose superior invasive ability has been already demonstrated [23–25] as compared to BxPC3 and MiaPaca cells.

In PC patients, the univariate analysis showed a protective role, in terms of reduced mortality, of high DNMT3B expression levels in noninvasive tumours, whereas in invasive tumours such an effect for DNMT3B overexpression was not further observed.

Moreover, in our *in vitro* models of PC cells, PPAR*γ* displayed higher levels in PANC-1 cells as compared to the other cell lines whilst CFPAC and MiaPaca cells presented the lower PPAR*γ* mRNA levels. DNMT1 showed the same trend of PPAR*γ* corroborating the positive correlation between PPAR*γ* expression with DNMT1 in PC tissue.

When treated with PPAR*γ* ligand (rosiglitazone), pancreatic cancer cells responded in different manner suggesting a dose-dependent and time-dependent effect. The observed difference of PPAR*γ* and DNMTs levels and the differences in rosiglitazone response in the different cell lines could be due to their different genetic background [23]. Moreover, DNMTs alterations triggered by a low dose of rosiglitazone after 24 hours, which surprisingly was not observed upon incubation with a higher dose for 48 hours, indicate the existence of cell adaptative mechanisms to enhanced and perhaps saturated PPAR signaling.

## 5. Conclusion

Our study demonstrated that PPAR*γ* positively correlates with DNMT1, but not with DNMT3B, and that higher DNMT3B mRNA levels in presence of noninvasive tumor predict longer survival in pancreatic cancer patients whereas in presence of invasive tumour higher DNMT3B mRNA levels were associated with a poor prognosis.

## Figures and Tables

**Table 1:** Clinical and pathological features of patients with pancreatic ductal adenocarcinoma (PDAC).

	PDAC
	*n* = 30
Age at diagnosis, median (*Q*1–*Q*3)	69 (42–81)
Duration of follow-up, median (*Q*1–*Q*3)	18.48 (8.16–53.11)
Gender, male/female (%male)	25/5 (83)
Tumour localization, *n* (%)	
Head	28 (93)
Body-tail	2 (7)
Tumour type, *n* (%)	
Adenocarcinoma	24 (80)
Adenocarcinoma mucinous	6 (20)
Tumour grading, *n* (%)	
G1: well differentiated	4 (13)
G2: moderately differentiated	16 (54)
G3: poorly differentiated	10 (33)
T: Tumour size, *n* (%)	
T2	5 (17)
T3	25 (83)
N: regional lymph nodes, *n* (%)	
N0	7 (23)
N1	23 (77)
Lymph nodes ratio, median (*Q*1–*Q*3)	0.13 (0.00–0.80)
Tumour stage, *n* (%)	
IIA	7 (23)
IIB	23 (77)
Perineural invasion, *y*/*n* (% *y*)	9/21 (30)
Margins of resection, *n* (%)	
R0: negative rection margins	22 (27)
R1: microscopic positive resection margins	8 (73)

Pancreatic cancer staging. “Exocrine and endocrine pancreas.” In [25].
